# They Might Cut It—Lysosomes and Autophagy in Mitotic Progression

**DOI:** 10.3389/fcell.2021.727538

**Published:** 2021-08-13

**Authors:** Saara Hämälistö, Jonathan Stahl-Meyer, Marja Jäättelä

**Affiliations:** Cell Death and Metabolism, Center for Autophagy, Recycling and Disease, Danish Cancer Society Research Center, Copenhagen, Denmark

**Keywords:** autophagy, cathepsin B, chromosome segregation, lysosome, mitosis, spindle

## Abstract

The division of one cell into two looks so easy, as if it happens without any control at all. Mitosis, the hallmark of mammalian life is, however, tightly regulated from the early onset to the very last phase. Despite the tight control, errors in mitotic division occur frequently and they may result in various chromosomal instabilities and malignancies. The flow of events during mitotic progression where the chromosomes condensate and rearrange with the help of the cytoskeletal network has been described in great detail. Plasma membrane dynamics and endocytic vesicle movement upon deadhesion and reattachment of dividing cells are also demonstrated to be functionally important for the mitotic integrity. Other cytoplasmic organelles, such as autophagosomes and lysosomes, have until recently been considered merely as passive bystanders in this process. Accordingly, at the onset of nuclear envelope breakdown in prometaphase, the number of autophagic structures and lysosomes is reduced and the bulk autophagic machinery is suppressed for the duration of mitosis. This is believed to ensure that the exposed nuclear components are not unintentionally delivered to autophagic degradation. With the evolving technologies that allow the detection of subtle alterations in cytoplasmic organelles, our understanding of the small-scale regulation of intracellular organelles has deepened rapidly and we discuss here recent discoveries revealing unexpected roles for autophagy and lysosomes in the preservation of genomic integrity during mitosis.

## Introduction

**FIGURE 1:**
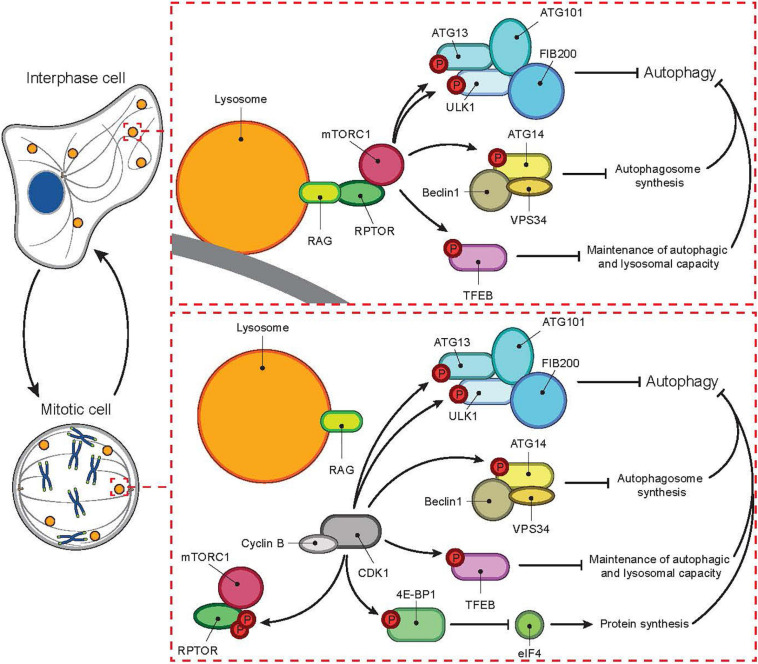
Cell cycle-dependent switch of autophagic master regulator. In interphase, autophagy is tightly controlled by mTORC1. In the absence of nutrients mTORC1 is inactive while autophagy is active. During nutrient-rich conditions, mTORC1 localizes to the lysosomal membrane and gets activated through an interaction between RPTOR and RAG proteins. Activated mTORC1 then inhibits autophagy at several levels, for example by phosphorylating ATG13, ULK1, ATG14, TFEB, and 4E-BPI. In mitosis, mTORC1 activity is inhibited even in the presence of nutrients by CDK1-mediated phosphorylation of RPTOR. In parallel, CDK1 overtakes mTORC1’s role as the inhibitor of autophagy by phosphorylating autophagic mTORC1 substrates listed above.

It is now nearly two decades from a dogma-creating report describing a dramatic reduction in the number of autophagosomes and a global shut-down of the autophagic machinery during mitotic phases of the cell cycle, from prometaphase until the initiation of telophase (Eskelinen et al., 2002). Upon the instigation of mitosis, the mammalian target of rapamycin complex 1 (mTORC1), which keeps the autophagic machinery silent in nutrient rich interphase cells, is shut down by cyclin-dependent kinase 1 (CDK1)-mediated phosphorylation of regulatory-associated protein of mTOR (RPTOR) (Odle et al., 2020). Simultaneously, CDK1 overtakes mTORC1’s role as the key inhibitor of autophagy by phosphorylating Unc-51 like autophagy activating kinase (ULK1), autophagy-related protein (ATG13) and other autophagic substrates of mTORC1 (Figure 1, adapted from Odle et al. (2020)). Thus, the view of drastically inhibited bulk autophagy during mitosis still holds, while emerging data is shedding light on the importance of selective autophagic and lysosomal processes for the accurate progression of mitosis (Holdgaard et al., 2019; Hämälistö et al., 2020; Odle et al., 2020; Almacellas et al., 2021). The role of lysosomal pathways in mitosis is further supported by the frequent appearance of nuclear abnormalities and accumulation of midbody remnants, both characteristics of a non-functional cell division machinery, in cells from patients with genetic disorders of lysosomal function (Kanazawa et al., 2000; Pohl and Jentsch, 2009). These findings stress the importance of coordinated enzymatic activities of cytoplasmic organelles in relation to a functional cell cycle. Although the idea of lysosomal contribution to the cell cycle control has already been presented in the 1960s, the study of lysosomal pathways has until recently focused mostly on interphase cells and the role of lysosomes in providing building blocks for DNA replication in the S phase of the cell cycle (Vanzo et al., 2020; Merchut-Maya and Maya-Mendoza, 2021). Here, we review the recent data on changes on lysosomes and the autophagic machinery during the cell cycle and their newly discovered roles in the control of cell division.

## Lysosomes Rewire at Early Mitosis

Mitosis starts by the activation of CDK1, an essential regulator that phosphorylates nearly 1,000 proteins including Polo-like kinase 1 and Aurora B (Crncec and Hochegger, 2019). This phosphorylation cascade initiates the rapid nuclear envelope break-down, spindle pole formation, cell rounding and finally the alignment of chromosomes, all of which only takes 10–20 min (Crncec and Hochegger, 2019; Dey and Baum, 2021). While the above-mentioned mitotic events are thoroughly depicted, the role of cytoplasmic organelles in this process is only beginning to emerge (Stahl-Meyer et al., 2021).

**FIGURE 2:**
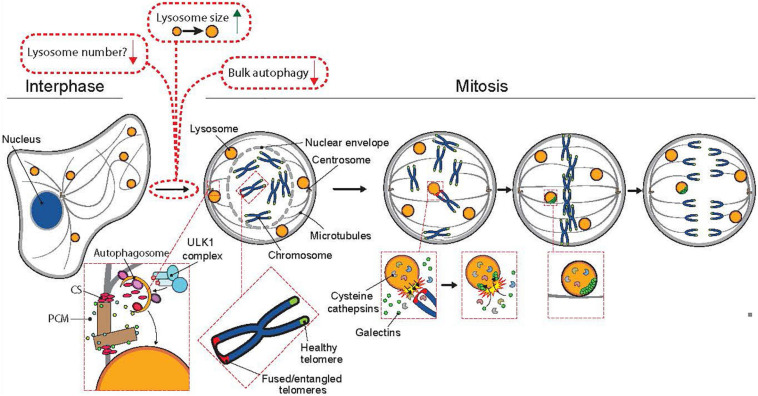
The involvement of lysosomes and autophagy in mitosis. Mitotic cells have reduced autophagic activity and fewer but larger lysosomes than interphase cells. Upon entry to mitosis, bulk autophagy is shut down by CDK1 (see Figure 1 for additional information). In prophase, doryphagy, a type of selective autophagy targeting the centriolar satellites (CS), maintains centrosome integrity, which ensures the formation of a bibolar spindle. The breakdown of the nuclear envelope marks the onset of prometaphase, where the chromosomes can interact with the kinetochore-microtubules of the mitotic spindle. In prometaphase, a few lysosomes are recruited to the proximity of chromosomes with unresolved telomere fusions or entanglements, where their limiting membrane is transiently permeabilized resulting in the release of lysosomal cathepsins into the chromatin and entrance of galectins (marker proteins for lysosomal membrane permeabilization) into the lysosomes. Cathepsin B-mediated cleavage of histone H3 assists the segregation of the fused or entangled chromosomes in anaphase thereby preventing chromosome segregation errors, micronuclei formation and chromosomal instability.

In interphase cells, lysosomes move constantly between two spatially distinct pools, the main one assembled around the microtubule-organizing center (MTOC), and the other one in the close proximity of the plasma membrane (Matteoni and Kreis, 1987). The bidirectional movement of lysosomes between these pools is relatively fast and occurs along the microtubule tracks for example in response to starvation and growth factor signaling, which trigger inward and outward trafficking, respectively (Colaco and Jäättelä, 2017). When cells round up at early mitosis, the interphase microtubules reorganize in a step-wise manner to form the mitotic spindle (Mchedlishvili et al., 2018); concomitantly with the microtubule reshaping, the lysosomes are dispersed throughout the cytoplasm until they rapidly recluster around the MTOCs in telophase (Matteoni and Kreis, 1987). It is unclear whether the movement of mitotic lysosomes is actively regulated for example by motor proteins on the mitotic spindle or whether it occurs merely by random diffusion. Recent studies have, however, demonstrated that a subpopulation of lysosomes dock at the close vicinity of prometaphase and metaphase chromosomes (Hämälistö et al., 2020; Almacellas et al., 2021). Some of these chromatin-proximal lysosomes contain cytosolic galectins, which is indicative of a recent lysosomal membrane permeabilization and release of lysosomal hydrolases (Aits et al., 2015). Based on high resolution microscopy images, the majority of galectin-positive leaky lysosomes are in direct contact with the telomeric regions of the chromatin and the surrounding chromatin is decorated by the active form of a lysosomal cysteine protease cathepsin B (CTSB) (Hämälistö et al., 2020; Figure 2). As described in more detail later in this review, such localization of leaky lysosomes appears to play an essential role in the further progression of mitosis.

In addition to changes in their localization, mitotic lysosomes have been reported to be larger in size and fewer in number than interphase lysosomes (Almacellas et al., 2021). The reasons for these changes in mitosis are not well understood, while in general, the increased lysosomal size correlates with less acidic lumen and is characteristic for certain lysosomal storage diseases with reduced lysosomal activity (de Araujo et al., 2020). Even though the lysosomal size is increased in healthy mitotic cells, uncontrolled growth of lysosomes is detrimental. The greatly enlarged late endosomes and lysosomes in cells lacking the transport protein myosin 5b disrupt epithelial integrity by physically inhibiting appropriate mitotic spindle orientation (Leng et al., 2019).

While our understanding of the role of lysosomes in the division of mammalian cells is still limited, we can get inspired by studies in budding yeast *Saccharomyces Cerevisiae*, where increased size and MTORC1 activity of the vacuole (yeast lysosome) are required for the exit from the G1 phase of the cell cycle and the budding of daughter cells, respectively (Jin and Weisman, 2015). Furthermore, the vacuolar pH of the mother cell increases progressively in each cell division until the vacuole fails to provide amino acids to maintain functional mitochondria and the cell fails to bud further (Hughes and Gottschling, 2012). It remains to be studied whether the lysosomal size and acidity controls also the division of mammalian cells by regulating for example the flow of amino acids, lysosomal signaling, or lysosomal membrane integrity.

## Targeted Autophagy Stabilizes Centrosomes

As prophase progresses to prometaphase, the nuclear envelope dismantles, microtubules are extended to the exposed chromatin and the centrosomes, the nucleation sites from where the tubulin filaments grow and travel to the opposing ends of the chromatin to form the mitotic spindle (Dey and Baum, 2021). Centrosomes serve as the main microtubule-organizing centers of the cells and several autophagy-regulating proteins including microtubule-associated proteins 1 light chain 3B (LC3B), gamma-aminobutyric acid receptor-associated protein (GABARAP), sequestesome-1 and WD repeat domain phosphoinositide-interacting protein 2 (WIPI2) and have been detected at the centrosome region in mitotic cells (Joachim and Tooze, 2017; Holdgaard et al., 2019). Interestingly, a recent study has revealed a new type of selective autophagy, doryphagy, that targets at least two essential centriolar satellite organizers, pericentriolar material 1 protein (PCM1) and centrosomal protein of 131 kDa (CEP131), and thereby preserves centrosome organization and stability (Holdgaard et al., 2019). Accordingly, the inhibition of autophagy by the depletion of key autophagy factors (e.g., ULK1, ATG5, and ATG7) results in the accumulation of large abnormal centriolar satellites and disturbs the balance of the mitotic spindle in such a way that the centrosomes dismantle upon nuclear envelope break-down, which results in the collapse of the mitotic spindle and eventually in chromosomal instability or cell death (Holdgaard et al., 2019; Figure 2, adapted from Stahl-Meyer et al. (2021)). Because both chromosomal instability and alterations in centrosome structure and number are linked to tumorigenesis, this specific type of autophagy may contribute to the tumor suppressive function of autophagy. Even though doryphagy affects the cell division, it occurs during the interphase and may also affect the centrosome-dependent trafficking of endolysosomal vesicles along the tubulin cytoskeleton in interphase cells. In line with this, disturbed centrosome homeostasis in pancreatic cancer cells has recently been linked to lysosomal dysfunction, release of small extracellular vesicles and increased invasive activity (Adams et al., 2021).

## The Separation and the Final Cut: Vesicles on the Move

When cells have aligned their chromosomes properly along the metaphase plate, the anaphase-promoting complex (APC) initiates the process of sister chromatid separation (Crncec and Hochegger, 2019). In addition to the above-described role of doryphagy in the formation of the mitotic spindle, accumulating evidence suggest that lysosomes are required also for the actual separation of sister chromatids. RNAi-mediated depletion of the major lysosomal protease CTSB or microtubule-dependent lysosome transport proteins, kinesin-1 heavy chain (KIF5B), ADP-ribosylation factor-like protein 8B (ARL8B) or pleckstrin homology domain-containing family M member 2 (PLEKHM2), significantly delays the passage of cells through the metaphase; furthermore, they disturb the segregation of chromosomes in anaphase resulting in significant increases in chromatin bridges, lagging chromosomes and micronuclei (Hämälistö et al., 2020; Almacellas et al., 2021). Severe chromosome segregation phenotypes were observed in cells where CTSB was inhibited pharmacologically upon entering mitosis suggesting that CTSB activity has a direct role in mitotic chromosome segregation (Hämälistö et al., 2020; Almacellas et al., 2021). This view is further supported by the demonstration of extralysosomal CTSB activity on metaphase chromatin, CTSB-mediated cleavage of histone H3 in (pro)metaphase and appearance of segregation errors in cells expressing CTSB resistant histone H3 mutants (Hämälistö et al., 2020). Although the exact mechanism by which this occurs in the mitotic cells remains to be elucidated, the cleavage of Histone-3 by CTSB, but not that of H2B, seems to promote a successful chromosome segregation. Other putative CTSB substrates in early mitosis include two cohesin-binding proteins, wings apart-like protein homolog (WAPL) and sister chromatid cohesion protein PDS5 homolog B (PDS5B), both of which accumulate in mitotic cells when lysosomal hydrolases are inhibited (Almacellas et al., 2021). The cohesins are needed in metaphase to keep the sister chromatids together until the anaphase-promoting complex (APC/C) degrades securin, the inhibitor of the cohesion-destabilizing separin. This leads to degradation of a key protein Scc1/Rad21 and finally cohesin release and segregation of the sister chromatids (Dey and Baum, 2021). It should be noted here that the necessity of cathepsin-mediated cleavage of WAPL and PDS5B for proper chromosome segregation has not yet been experimentally validated. The extralysosomal cathepsin activity during metaphase is, however, emerging as a novel check point controlling the transit of cells from metaphase to telophase. Nevertheless, it remains unclear when the chromosome segregation requires the assistance of extralysosomal cathepsins. The high abundance of telomeric sequences in chromatin bridges and lagging chromosomes observed in the absence of cathepsin activity suggest that lysosomal hydrolases may be called to resolve telomere fusions and entanglements that are relatively common in dividing cells. This notion is further supported by the colocalization of mitotic leaky lysosomes with telomeres and their dramatic increase in cells with excessive formation of telomere fusions due to the lack of telomeric repeat-binding protein 2, which is responsible for the formation of the telomere protecting t-loop structures (De Lange, 2018; Hämälistö et al., 2020). Thus telomere fusions or entanglements may, by an yet unknown mechanism, trigger the leakage of a proximal lysosomes, which then releases CTSB and other lysosomal hydrolases to the problem site.

In the last phase of mitosis, telophase, the divided genetic material is being re-enveloped inside the nuclear lamina. At this time, the daughter cells elongate with the help of their reestablished actin and tubulin cytoskeletons, grow their plasma membrane to supply the cellular division and reactivate the autophagosome formation (Eskelinen et al., 2002; Dey and Baum, 2021). Interestingly, the mitotic “leaky” lysosomes detected in the earlier mitotic phases appear to be sorted to the daughter cells in a random manner and eventually re-gain their interphase-like morphology (Hämälistö et al., 2020). Lysosomal clustering at the intercellular bridge has been suggested to promote the activities at the cleavage furrow (Rajamanoharan et al., 2015). Recent data indicate that phosphoinositol-4-kinase-dependent lysosome exocytosis provides membranous material to the furrow, and inhibiting this process causes mitotic failures (Helassa et al., 2019). Before the final cut, the portioning of endolysosomal vesicles into daughter cells occurs in an ordered, yet imprecise, manner. In other words, the division is similar to a stochastic distribution of the organelles into two compartments and results in comparable organelle copy number in daughter cells (Bergeland et al., 2001). Furthermore, the endosomal vesicles and their respective expression levels have been shown to affect Notch signaling in the unequally sized daughter cells, with direct implications on development and physiology (Daeden and Gonzalez-Gaitan, 2018). In cytotoxic CD8 + T cells, the random partitioning of lytic granules/lysosomes to daughter cells in telophase leads to differential lytic capabilities upon target engagement in the cells (Lafouresse et al., 2021). Intriguingly, it was recently shown that mitochondria have parallel mechanisms with the actin cytoskeleton ensuring a targeted distribution of mitochondria to the dividing daughter cells (Moore et al., 2021).

Ultimately, the actin tubulin-rich midbody ring that forms in between the daughter cells in telophase needs to be cleared away. In this process, the autophagy regulators such as sequestesome-1 and LC3 interact with the midbody ring structure and help to clear the area from dense midbody remnants (Pohl and Jentsch, 2009). This is evident in lysosomal storage disorders (LSD), many of which are characterized by the accumulation of midbody structures (Pohl and Jentsch, 2009). Failure to clear the midbody leads to the accumulation of midbody remnants and unwanted signaling in the cells: cancer promoting signaling, cell polarity changes and modulation of intra- and intercellular signaling has been reported as a result of derailed midbody clearance (reviewed in Antanavičiūtė et al., 2018). The cells with chronic lysosomal impairment also accumulate micronuclei, which are common markers of chromosomal instability. For example, rapidly proliferating tissues such as intestinal crypts and the skin epidermis from cathepsin B deficient mice have significantly increased numbers of micronuclei (Hämälistö et al., 2020). Lysosomal dysfunction may also lead to the formation of other nuclear abnormalities, including toroidal nuclei characterized by a distinct chromatin-free region in the middle of the nucleus (Almacellas et al., 2021).

## Difficulties to Divide: Examples of Lysosomal Storage Diseases

The elegant balance of lysosomal enzyme activities and their effect on various aspects of cell division can be appreciated in lysosomal storage disorders such as the neurological Krabbe disease (also known as globoid cell leukodystrophy). Here, the lysosomal enzyme galactosylceramidase is functionally deficient, which results in the accumulation of its metabolite galactosylsphingosine (psychosine) (Kanazawa et al., 2000). The exposure of human leukemia and cervical cancer cells to psychosine leads to failed cytokinesis due to, at least in part, deregulated actin dynamics in the late stages of mitosis. This results in the formation of multinucleated giant cells, which may be related to the multinucleate cellular phenotype characteristic of the Krabbe disease (Kanazawa et al., 2000). Also many other lysosomal storage diseases, e.g., Niemann-Pick C1 that is caused by a genetic defect in lysosomal cholesterol export and infantile neuronal ceroid lipofuscinosis caused by reduced lysosomal palmitoyl protein thioesterase, are characterized by aneuploid and multinucleate cells indicating serious problems in cell division (Gupta et al., 2003; Granic and Potter, 2013). Taken together with the experimental data presented earlier, the accumulation of nuclear abnormalities in patients with various lysosomal defects underlines the importance of an unexpected link between healthy lysosomes and cell division.

## Conclusion and Perspectives

*Omnis cellula e cellula*—all cells come from cells. A quote from the German physician Rudolf Virchow from 1,855 stating the fact that cells arise from the growth and division of existing cells and the hand-over of the genetic and cytoplasmic information to the next generation. Despite the intense research over decades to decipher the molecular infrastructure of this process, many levels of regulation are still to be elucidated. The cell cycle is quality-checked at specific checkpoints and accumulating data describe new levels of regulation to this process. In addition to the lysosome-dependent regulation of mitosis described above, emerging evidence exists to stress the importance of autophagy regulators in various cell cycle phases: recently, the activating molecule in BECN1-regulated autophagy protein 1 (AMBRA1) was demonstrated to control cyclin D levels and genomic integrity during S phase (Maiani et al., 2021). Additionally, a recent paper elegantly illustrates the actin-driven targeting of mitochondria in cytokinesis—a fine example of how, in addition to the chromatin, the cytoplasmic organelles are directed to the daughter cells in a way that supports high fidelity in the progeny cells (Moore et al., 2021). Similar mechanisms may contribute to the random but equal distribution of lysosomes in cell division. It is clear that our understanding of the role of lysosomal pathways in cell division is only in its infancy. The recent discoveries of novel roles for interphase lysosomes for example in cellular metabolism, invasion, calcium signaling, pH control and cell death, together with the rapidly developing technologies allowing more and more precise studies of individual lysosomes in real time (Ellegaard et al., 2021; Zhu et al., 2021) will hopefully pave the way to new results explaining the molecular basis of the lysosomal control of cell division described above. The generation of multinucleated cells in the lysosomal storage disorders and the impact these have on the disease progression, has not been characterized. A detailed analysis of these multinucleate cell populations and their possible targeting in the storage diseases may be worth studying. This could lead to new unexpected discoveries linking lysosomal pathways to the cell division machinery and help design new diagnostic tools and therapies for various conditions with compromised lysosomal function and defected cellular division.

## Author Contributions

SH wrote the first draft of the manuscript. JS-M commented the text and prepared the figures. MJ guided the process and wrote the final version of the text. All authors contributed to the article and approved the submitted version.

## Conflict of Interest

The authors declare that the research was conducted in the absence of any commercial or financial relationships that could be construed as a potential conflict of interest.

## Publisher’s Note

All claims expressed in this article are solely those of the authors and do not necessarily represent those of their affiliated organizations, or those of the publisher, the editors and the reviewers. Any product that may be evaluated in this article, or claim that may be made by its manufacturer, is not guaranteed or endorsed by the publisher.
